# Integrating model systems and genomic insights to decipher mechanisms of cancer metastasis

**DOI:** 10.1038/s41576-025-00825-2

**Published:** 2025-03-10

**Authors:** Michelle M. Leung, Charles Swanton, Nicholas McGranahan

**Affiliations:** 1 Cancer Research UK Lung Cancer Centre of Excellence, University College London Cancer Institute, London, UK; 2 Cancer Genome Evolution Research Group, Cancer Research UK Lung Cancer Centre of Excellence, University College London Cancer Institute, London, UK; 3 Cancer Evolution and Genome Instability Laboratory, The Francis Crick Institute, London, UK; 4 Department of Oncology, University College London Hospitals, London, UK

## Abstract

Deciphering metastatic processes is crucial for understanding cancer progression and potential treatment options. Genetic studies of model systems engineered to mimic metastatic disease, including organoids, genetically engineered mice and human cell lines, have had an important role in shaping our understanding of the metastatic cascade and how it can be manipulated. More recently, advances in high-throughput sequencing have enabled human metastases to be studied at single-cell and single-nucleotide resolution, providing insights into metastatic evolution and phenotypes of both cancer cells and immune cells. However, human tissue studies are often correlative and descriptive, whereas experimental models are reductionistic by nature, meaning that individual results should be interpreted with caution. Crucially, these seemingly disparate branches of metastasis research can and should complement each other to strengthen and validate findings. Here, we explore the synergies between model systems and sequencing studies and outline key areas that must be explored to improve our understanding of the metastatic process.

## Introduction

**Figure 1:**
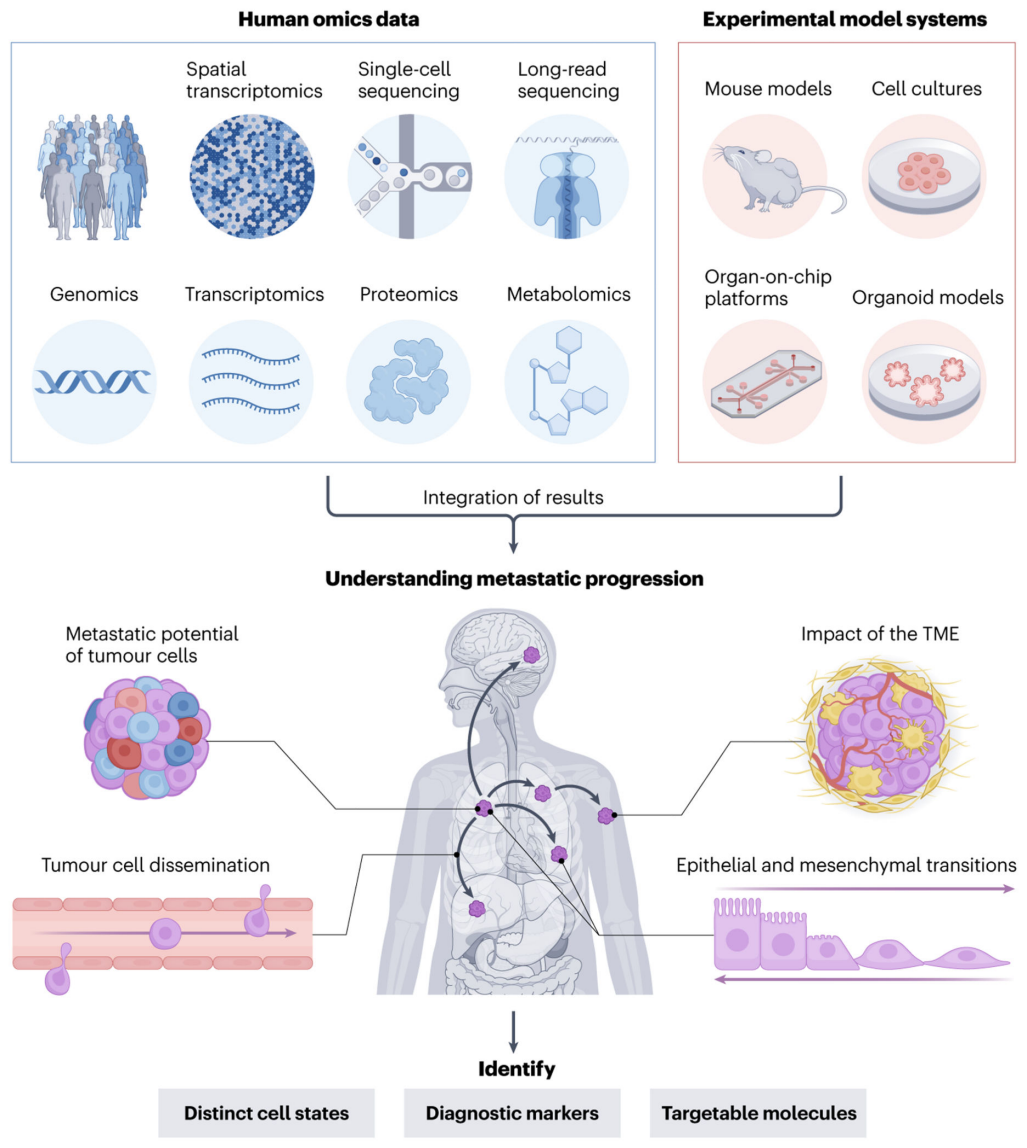
Overview of the synergism between experimental models and omics analyses in metastasis research. Advances in metastasis research can be made by combining analytical results from omics data obtained from human cohorts — ranging from genomics and transcriptomics (involving long-read DNA sequencing, single-cell RNA sequencing and spatial transcriptomics) to proteomics and metabolomics — with functional results from experimental systems, including (genetically engineered) mouse models, cell cultures, organoid models and organ-on-a-chip platforms. This integration between different approaches allows for key factors in determining metastatic progression — the metastatic potential of tumour cells, epithelial and mesenchymal transitions, the impact of the tumour microenvironment (TME) and tumour cell dissemination — to be better understood, with the aim of identifying targets for better disease prevention, detection and treatment.

Most cancer-related deaths can be linked to metastasis^1,2^, the process by which cancer cells move beyond the constraints of their initial tumour site and colonize other tissues^3,4^. Metastasis typically involves the key stages of invasion, intravasation, circulation, extravasation and colonisation^1–6^, whereby tumour cells gain an invasive phenotype, enter and travel through the bloodstream, then invade a distant organ site to form the subsequent metastasis^7^. A deeper understanding of the processes underlying metastasis — how cancer cells deviate from normal mechanisms of growth, motility and interactions with the environment — could illuminate potential targets and timepoints for metastatic disease detection, intervention and treatment, whilst also providing insights into the ‘normal’ biology of the cell types involved (Fig. 1).

Experimental systems such as organoids, genetically engineered mice, human cell lines and organ-on-a-chip platforms have helped to elucidate the interlocking, sequential set of processes that contribute to the metastatic cascade^7^. These approaches illustrate not only the cancer cell-intrinsic changes that occur during metastasis but also their interplay with extrinsic factors, such as cells of the immune system and the extracellular matrix (ECM). Many findings from experimental models support the ‘seed and soil’ theory of metastasis^8^, which proposes that tumour cell ‘seeds’ require a suitable microenvironment or ‘soil’ for metastatic growth^9^.

More recently, advances in high-throughput omics technologies, ranging from genomics and transcriptomics to proteomics and metabolomics, have allowed for the study of cancer and its metastases from human tissue samples at increasingly high resolution^10–16^. In particular, genomic analyses have provided evidence to support the theory that cancer, and hence metastasis, is an evolutionary process^17^. Through multi-omics studies (for example, DNA and RNA sequencing from the same sample) and paired analysis of human primary tumours and their respective metastases, we are now able to estimate when in molecular time metastases occur, their clonal origins and the molecular relationships between two metastases, as well as between a metastasis and its primary tumour^10,18,19^. These characteristics of a tumour could be predictive of disease progression.

Metastasis research, therefore, like the disease itself, is on a course of evolution, in which the experimental model approaches and the analysis of human data through increasingly advanced sequencing technologies are developing in parallel but can also complement each other. For example, the omics approaches that are being applied to increasingly large cohorts of human data can fill the knowledge gaps left by model studies and, in turn, raise new questions. Conversely, the omics data, which are largely descriptive, require experimental validation in appropriate model systems to verify the findings and determine the individual contributions of specific factors to metastasis.

This Review explores the interplay between experimental models of metastasis and the analysis of human metastatic samples by sequencing technologies, using broad examples from our existing knowledge of cancer metastasis. For the main processes of metastatic progression, we consider the benefits of and key, representative, insights gained from each approach. We evaluate knowledge gaps that require further study and discuss discrepancies in the results from experimental models and genomic approaches that will need to be clarified to better understand the complex process of metastasis.

### The ‘metastatic potential’ of tumours

Not all tumours metastasise, making it important to delineate non-metastasising cells from those that have the ‘potential’ to metastasise. The hallmarks of metastasis, which are key to successful colonisation, include increased motility and the ability to modulate the microenvironment^20^.

#### Heterogeneity of metastases

Model systems have been extensively used to study the role of increased cell motility in promoting the metastatic phenotype, particularly in ‘simple’ organisms such as *Caenorhabditis elegans*, owing to their ease of manipulation and visualisation^21^. Despite the lack of lymphatics and blood vessels in some of these simple organisms, which therefore do not fully recapitulate the spread of human metastatic cancer, many fundamental mechanisms underlying the biology of metastatic cells have been revealed. For example, live imaging of *C. elegans* revealed that cells invade the basement membrane **[G]** in an early step towards metastatic invasion and migration^22^. In a separate *C. elegans* study, it was shown that an invasive phenotype, including invadopodia **[G]** formation, is associated with G1 cell cycle arrest^23^. This ‘cost’ of invasion implies that metastatic cells can either “go or grow”^21^; as such, there is an increased likelihood of successful invasion when clusters of cells migrate collectively^24,25^, whereby ‘leaders’ and ‘followers’ have distinct phenotypes (invasive versus proliferative) in support of each other. In an *in vitro* culture system of squamous cell carcinoma cells, collective migration was aided by fibroblast-mediated remodelling of the ECM, forming ‘tracks’ for cancer cells to follow^26^, which emphasises the role of the tumour microenvironment (TME; reviewed in a later section) in determining metastasis. The increased metastatic potential of heterogeneous cell groups as indicated by these experimental models is supported by the genomic analysis of paired human samples from primary tumour and metastasis, which highlights that metastases are often polyclonal^14,27,28^, composed of genetically heterogeneous populations of cancer cells — reflective of multiple seeding events or genetically distinct cells travelling together.

#### Genomic instability of metastases

Studies involving large cohorts of bulk or single-cell genomic sequencing data^10,16,29–36^ have emphasised the role of genomic instability in the acquisition of metastatic potential. For example, in a pan-cancer cohort of more than 25,000 individuals with metastatic disease^29^, there was an increase in the number of whole-genome doubling events and copy number alterations in metastases across multiple cancer types when compared to primary tumours of the same type. These studies also indicate that there is limited evidence for universal genetic drivers of metastasis that always and only occur at the point of metastatic transition. Rather, driver events that confer a fitness advantage in primary tumours are also frequently selected for at metastatic sites. For example, genetic driver events that contribute to a more aggressive phenotype, such as *MYC* amplifications, *TP53* mutations and *CDKN2A* deletions, are enriched in metastatic cohorts. However, it is of note that most of these large cohort studies have analysed unpaired primary and metastasis samples^12,29,37^; analysing cohorts of paired primary–metastasis samples could distinguish between driver alterations that occur in the primary tumour and may increase the propensity for metastasis and truly metastasis-unique genetic driver events. For example, metastasis-unique mutations in lung cancer-associated genes were seen in 33% of samples in the TRACERx (TRAcking Cancer Evolution through therapy (Rx)) non-small cell lung cancer (NSCLC) cohort^10,38,39^.

A role for genomic instability in the initiation of metastasis as indicated by sequencing studies has been examined in greater detail *in vivo*. In a mouse model of human triple-negative breast cancer **[G]**, it was observed that a gene signature of high chromosomal instability correlates with the expression of genes involved in epithelial–mesenchymal transition **[G]** (EMT)^40^, a process that mediates the migratory and invasive behaviour of metastatic cells (reviewed in the next main section). It is proposed that chromosomal mis-segregation may lead to the release of DNA into the cytosol of tumour cells, triggering activation of the cGAS–STING innate immune signalling pathway, which leads to an inflammatory response and the promotion of EMT^40^.

#### Patterns of dissemination

The relative molecular timing of metastatic spread is difficult to derive from experimental models owing to the potential dormancy of disseminated tumour cells (reviewed in a later section) but it can be inferred through sequencing of human tumour samples. One method involves comparing somatic genetic variants between paired primary and metastatic tumour samples^10^. The timing of metastasis is clinically relevant because metastases that disseminate early during tumour evolution would likely not share many mutations with the primary tumour, as both would continue to evolve after metastasis has occurred. This discrepancy between primary and metastatic tumours could render treatments less effective^17^ should targetable genetic alterations be subclonal or absent in the metastasis^10^.

Intuitively, different cancer types have different patterns of dissemination. For example, a study showed early metastatic dissemination (when the size of the primary tumour is below detection threshold) in 81% of individuals in a colorectal cancer cohort^41^ compared with only 25% of individuals in the multi-region TRACERx cohort of NSCLC^10^. Interestingly, in the same NSCLC study^10^, it was shown that sampling bias could lead to the erroneous classification of a tumour as having early dissemination; indeed, if the same calculations were repeated after downsampling to a single region of the tumour, 83% of late-disseminating NSCLC tumours were misidentified as early-disseminating. On the one hand, this comparison highlights the need for multi-region or representative sampling^42^ for a more accurate representation of intra-tumour heterogeneity. On the other hand, it illuminates the disparity in the field in terms of defining ‘early dissemination’ — through either physical tumour size or a molecular measure of genetic divergence. This is important to consider in comparative studies as large numbers of somatic mutations in metastases (which would define a tumour as late-disseminating in molecular terms) have been observed in small tumours (defined as early-disseminating in terms of size), as well as prior to tumour initiation^43–45^. Therefore, the inference of early dissemination based on tumour size can be compatible with the inference of late dissemination based on somatic mutations.

Beyond the timing of metastasis, it is also important to consider the site of metastasis (metastatic organotropism). For example, through the sequencing analysis of more than 2,000 lung adenocarcinoma samples, it was found that neoadjuvant therapy **[G]** was significantly associated with metastasis to the central nervous system, whereas an enrichment of *SMARC4* alterations in the primary tumour, likely disrupting chromosome accessibility and nucleosome occupancy, highly correlated with bone metastasis^33^. In addition, *SMARC4* inactivation in primary tumours from patients without metastasis at the time of initial sequencing was associated with decreased time from primary tumour to bone metastasis^33^. Patterns of metastatic organotropism revealed by sequencing studies can be recapitulated in experimental systems to provide deeper mechanistic insights; for example, barcoded breast cancer cell lines that were injected into immunodeficient mice could subsequently be traced to map their dissemination patterns^46^. Although this system, unlike the use of humanised mice^47^, is caveated by the lack of potential interactions between tumour cells and the immune system, it could pave the way for the creation of a ‘metastasis map’ to guide treatment and prevention.

#### Phylogeny of dissemination

Metastases can ‘seed’ from either a single subclone within the primary tumour (monoclonal dissemination) or from multiple subclones (polyclonal dissemination)^48^. These patterns can be visualised as tumour phylogenetic trees that represent the clonal temporal architecture of a person’s metastatic disease^48–51^. However, metastases can themselves seed further metastases^52–54^, adding another layer of complexity to metastatic migration. Computational and mathematical models are being developed to infer patterns of dissemination from genomic data^53^. Nonetheless, there are uncertainties associated with such predictions owing to insufficient sequencing depth, poor sample quality (for example, the use of autopsy tissue) and overall undersampling of the tumour(s). Indeed, it is clear that undersampling may limit the detection of polyclonal dissemination^10^. For example, the reanalysis of a human breast cancer cohort^55^ suggested that the original conclusion of a polyclonal dissemination pattern was likely incorrect and that monoclonal seeding was the more parsimonious solution^53^. In the future, increased spatial and temporal sampling of tumours, including autopsy studies and longitudinal sampling through analysis of circulating tumour DNA **[G]** (ctDNA)^56^, as well as the incorporation of other data types, such as epigenomics, transcriptomics and clinical imaging data, could improve the predictions of metastatic migration patterns from genomic studies.

**Figure 2:**
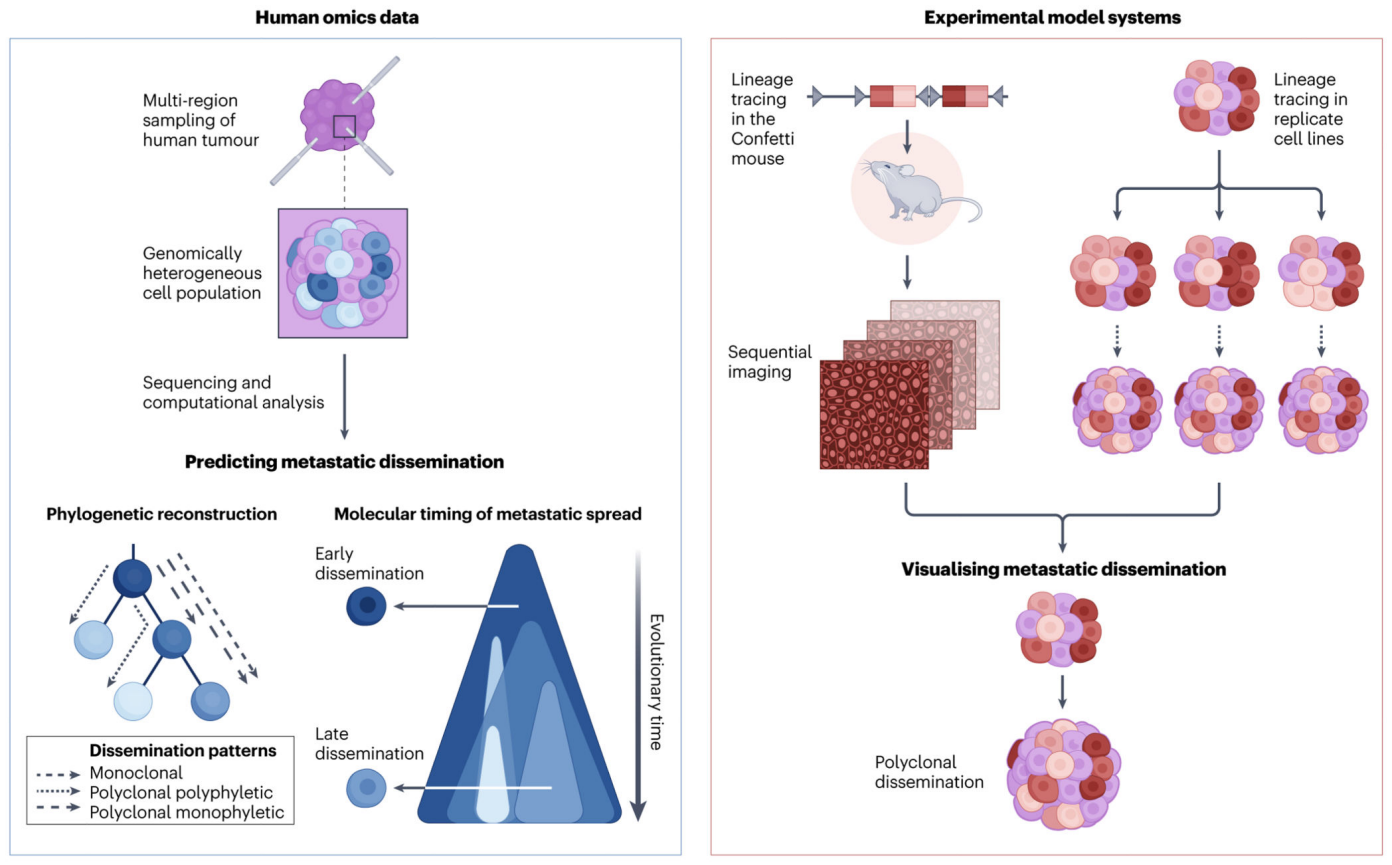
Predicting and visualising metastatic dissemination patterns from experimental models and human omics data. Metastatic dissemination patterns can be inferred and the evolutionary history and molecular timing of metastasis reconstructed using computational methods through sequencing of human samples. The accuracy of such inferences improves upon multi-region sampling. Lineage tracing in mouse models (such as the Confetti mouse) and in replicate cell lines, combined with sequential imaging, allows for the visualisation of these patterns of dissemination, providing evidence for previous inferences such as polyclonal dissemination.

The current human omics datasets do not capture the full span of metastatic disease, only snapshots of the tumour at the time of sampling. This limitation can be overcome using experimental model systems such as lineage tracing (Fig. 2). For example, through multicolour lineage tracing in the Confetti mouse **[G]**, sequential imaging observations of multicoloured metastases have supported a polyclonal dissemination hypothesis^57^. Other multicolour lineage tracing studies, in genetically engineered mouse models of breast cancer and pancreatic cancer metastases^24,58^, also showed polyclonal metastasis that did not result from serial seeding. Another experimental method is to use the CRISPR–Cas9 system to induce unique genetic ‘scars’ in single tumour cells, thereby enabling the high-resolution molecular recording of single-cell lineages^59–61^. Using this method, it was shown that lung metastases in a mouse model of sarcoma arose from a single primary subclone^60^. Subsequent single-cell sequencing of the metastases at multiple time-points enabled the phylogenetic reconstruction of metastasis, metastatic route tracing and the identification of genetic driver events^62^. Lineage tracing studies can also be used to explore the roles of contingent evolution **[G]** and convergent evolution **[G]** during cancer development^63^.

### Epithelial and mesenchymal transitions

EMT and mesenchymal–epithelial transition **[G]** (MET), which involve the dysregulation of normal morphogenetics^64^, are involved in almost all metastatic diseases during the processes of metastatic initiation and colonisation, respectively.

#### Epithelial–mesenchymal transition

Transforming growth factor-β (TGFβ) is one of the best-studied inducers of EMT in experimental model systems, being able to induce EMT in many types of epithelial cell *in vitro*^65^. In human lung cell cultures, this was observed in terms of the increased expression levels of mesenchymal cell state markers, such as vimentin, and increased migration through wound-healing and Matrigel invasion assays^66^. Bisulfite sequencing of human lung cell lines has suggested that TGFβ-induced EMT occurs through demethylation within the *SNAIL1* promoter. This epigenetic alteration leads to the overexpression of SNAIL1, an inducer of EMT^66^ that was previously found to repress the expression of E-cadherin, a key epithelial cell marker^67^.

External factors, including air pollutants, also contribute to EMT during metastasis^68,69^. Continuous exposure to fine particulate matter (of less than 2.5 μm diameter, known as PM2.5), both *in vivo* in mice and *in vitro* in a human lung cancer cell line, led to increased expression of genes involved in EMT, including those in the PI3K–AKT and JAK–STAT pathways, both of which are downstream effectors of epidermal growth factor receptor (EGFR)^68^. This signalling pathway is likely induced by proheparin-binding EGF-like growth factor (HB-EGF), which was expressed and secreted by macrophages^68^; recent evidence has shown that there is a sustained increase in macrophage infiltration of the lung after exposure to PM2.5^70^. These results, which are supported by transcriptomic data showing that macrophage recruitment genes are upregulated upon PM2.5 exposure, provide a molecular explanation for an observation first made in the 1980s that nitrogen dioxide (another form of air pollution) increases the frequency and efficacy of tumour metastasis to the lungs in mice^69^.

Intriguingly, there is limited evidence that EMT might be dispensable in certain cancer types. Two independent studies showed successful metastasis *in vivo* upon EMT inhibition, in mouse models of pancreatic cancer and breast cancer^71,72^. However, despite the lack of an effect of EMT on metastatic growth, EMT seemed to induce chemoresistance in both studies. This could suggest the need for EMT-targeted treatment in conjunction with chemotherapy^71,72^. Further longitudinal studies in individuals with or without adjuvant therapy, and using a wider range of EMT markers and/or single-cell RNA-sequencing (scRNA-seq) to more accurately define cell states, are needed to confirm such conclusions and to help identify potential therapeutic targets.

#### A transitional spectrum

It is now well accepted that the ‘transition’ between epithelial and mesenchymal states occurs across a spectrum, whereby cells are not necessarily fully polarised to either extreme. This has been observed in circulating tumour cells (CTCs; reviewed in a later section) and in tumours themselves^61,73^. Many of these cells have hybrid-like states *in vitro*, expressing varying levels of both traditional epithelial cell markers, such as E-cadherin, and mesenchymal cell markers, such as vimentin^73,74^. This implies the presence of multiple tumour cell subtypes with differing levels of plasticity and invasiveness, an idea that is supported by scRNA-seq of both human and mouse tumours^61,73,75^. Furthermore, using assay for transposase-accessible chromatin using sequencing (ATAC-seq), differential expression levels of epithelial and mesenchymal markers were shown to be associated with varying degrees of chromatin remodelling^73^. Together, these data from sequencing studies indicate that there is a continuum of cells undergoing EMT that was previously overlooked in experimental model systems. Moreover, omics analyses on human tissue from various cancer types show the enrichment of EMT genes at the primary tumour site compared with matched metastases^76–79^, which concurs with the suggested role of EMT in the early stages of metastasis rather than in the resulting metastatic tumour.

#### Beyond the spectrum

Beyond the EMT spectrum lies the concept of cancerous lineage plasticity, which is likely not solely underpinned by genetic factors^80^. This has been shown in a recent study of metastatic colorectal cancer (CRC) combining results from single-cell transcriptomics, multiplexed imaging and patient-derived organoids^81^. By integrating omics data with experimental models, the authors showed that human tumour cells acquire a conserved, fetal progenitor-like, plastic state during metastasis, a state that can differentiate into multiple lineages that are enriched in CRC metastases^81^. However, these non-canonical states were not present in mouse models of CRC, highlighting inter-species differences^81^. Differences in EMT programmes were also observed between data from patients and mouse models, as well as between data from patients and human cell lines, in a brain metastasis study^82^, which indicates the need for careful interpretation from experimental models to human cancer.

In a separate study of lung cancer metastasis, scRNA-seq of human tissue also suggested that developmental plasticity is recapitulated in metastatic cells^83^. These results accentuate the need to resolve both genotype and phenotype from the same cell, which is being enabled by technologies such as ‘Genotyping of Transcriptomes’^84^, allowing for the determinants of the phenotypic landscape to be explored. Intriguingly, a mouse model of lung cancer metastasis showed that tumours with varying degrees of plasticity have differential immune sensitivity. For example, tumours with high levels of *SOX9* expression (a marker of developmental plasticity) had greater resistance to infiltration by natural killer cells through expression of self-MHC class I molecules^83^, which also emphasises the role of the immune system in sculpting tumour phenotypes and metastatic potential.

### Impact of the tumour microenvironment

The TME, comprising blood vessels, extracellular molecules and a range of cell types, provides the ‘soil’ to the cancer cell ‘seed’^85–87^. At each stage of the metastatic cascade, variations of the TME (in terms of both existing resident cells and additionally recruited components) can have a role in shaping the phenotype of tumour cells. One important aspect of the TME is the tumour stroma, which includes mesenchymal stem cells, immune cells and fibroblasts^85,88,89^. Here, we focus on studies of cancer-associated fibroblasts (CAFs), neutrophils and tumour-associated macrophages (TAMs) that showcase recent developments in the integration of studies of experimental model systems and human tissue-based omics approaches.

#### Cancer-associated fibroblasts

CAFs, which are malignant derivatives of fibroblasts from various origins^90^, are heterogeneous populations of cells that often make up most of the tumour-surrounding stroma^91,92^. They are also key determinants of cancer progression, involved in processes such as epithelial differentiation, basement membrane formation and inflammatory regulation^91,93^. CAFs might also function as physical barriers for cancer cells against infiltrating immune cells^94^. These functions of CAFs are associated with increased metastatic potential and poor patient prognosis in multiple cancer types^92,95–97^.

Through a combination of immunohistochemistry and flow cytometry of patient tumour cells, as well as RT-PCR of CAFs from mouse models^98,99^, it was found that distinct CAF subtypes have different abundances across cancer types. The use of single-cell sequencing has enabled further characterisation of these subtypes^97,100–103^. For example, inflammatory CAFs with increased expression of matrix-remodelling genes such as those encoding matrix metalloproteinases **[G]** (MMPs) are present in multiple cancer types, including breast and bladder cancer^97,100,104,105^. The ability of CAFs to remodel the ECM through the secretion of cytokines and exosomes that can enter the circulation allows for pre-metastatic niche **[G]** formation in distant organs, effectively preparing the tissue for the arrival of disseminated tumour cells^106,107^. These CAFs also upregulate genes in pathways associated with EMT^91,100^ and inflammation, through the secretion of EMT-inducing factors, including TGFβ and IL-6^100^.

Metastatic fibroblasts (MAFs) are adapted to the specific metastatic tissue niche from which they arise, thereby aiding successful metastatic colonisation^108^. Together with CAFs, MAFs are important in establishing the pre-metastatic niche, which crucially involves immune modulation^107^. In a mouse model of lung metastasis, the expression of *COX2*, which encodes an enzyme involved in prostaglandin production, in lung-resident fibroblasts reprogrammed exogenous myeloid cells towards an immunosuppressive state^105^. *COX2* expression and prostaglandin E2 secretion also rendered dendritic cells dysfunctional^105^. These findings highlight the importance of crosstalk between cells in the tumour stroma for metastasis to occur successfully.

Interestingly, compared with CAFs in the primary tumour, MAFs in human ovarian cancer metastases have increased expression of soluble factors and complement genes^104^, which in turn promote tumour growth and angiogenesis through inflammation^104^, by means such as neutrophil accumulation, as seen *in vivo*^109^. Importantly, the autocrine CXCL12–CXCR4-mediated upregulation of MMP2 and MMP9 expression can subsequently induce invadopodia formation by cancer cells through actin remodelling, a process that has been observed in many *in situ* studies (reviewed in refs.^110,111^), which promotes metastatic migration and invasion.

#### Neutrophils

Neutrophils, which are key inflammatory cells and the most abundant type of innate immune cell in humans, have an active role in metastatic progression and have been extensively reviewed elsewhere^112–115^. Here, we reference key examples of studies in a range of experimental models that have been validated using human omics data.

Like CAFs and MAFs, neutrophils have also been linked to formation of the pre-metastatic niche^116,117^. For example, it was observed in a mouse model that the accumulation of neutrophils in the lung is required for the subsequent metastatic colonisation of breast cancer cells in the lung^118^. Moreover, *in vitro*, neutrophil-derived factors favoured the clonal expansion of metastasis-initiating cells from a heterogeneous population of cancer cells^118^. Similarly, in a mouse model of ovarian cancer, neutrophils facilitated tumour implants on the omentum^119^. Human tissue omics studies support the contribution of neutrophils to metastatic dissemination; for example, imaging mass cytometry and genomics of paired samples showed that metastasising NSCLC clones have markedly increased neutrophil infiltration compared with non-metastasising tumours^94^.

#### Tumour-associated macrophages

TAMs can be derived from tissue-resident macrophages within the tumour tissue or from circulating blood monocytes^120,121^. They were previously thought to have a polarised, pro-tumorigenic and immunosuppressive phenotype (referred to as M2-like on the basis of *in vitro* studies)^122,123^. However, extensive scRNA-seq of human and mouse model tumour tissues has shown that TAMs exist on a phenotypic spectrum with varying gene expression profiles, which have heterogeneous effects at each step of the metastatic cascade^82,124,125^.

Multiphoton microscopy of fluorescently labelled tumour cells and macrophages *in vivo* showed increased density of TAMs along the invasive outer margins of mammary tumours, both as single motile cells and in clusters^126^. Strikingly, tumour cells only intravasated when macrophages were present at blood vessel borders, with a macrophage found at most one cell diameter away from an intravasating tumour cell^126^. Importantly, the number of perivascular macrophages, not the number of blood vessels, was associated with the efficacy of tumour cell intravasation^126^. A recent study of colorectal cancer used scRNA-seq profiles to analyse the clustering of TAMs by location relative to the tumour; patients with TAMs at the tumour border tended to have high levels of microsatellite instability and tumour mutational burden, which suggests that they might be more responsive to immunotherapy^127^. Using mouse models of breast cancer and human breast cancer cell lines, it was also observed that TAMs and breast cancer cells form a positive-feedback loop that promotes further EMT of tumour cells and increased recruitment of macrophages^128^. Transcriptomic analyses linked this phenotype to worse patient prognosis across multiple breast cancer cohorts^128^.

#### Interactions between cells in the stroma

**Figure 3:**
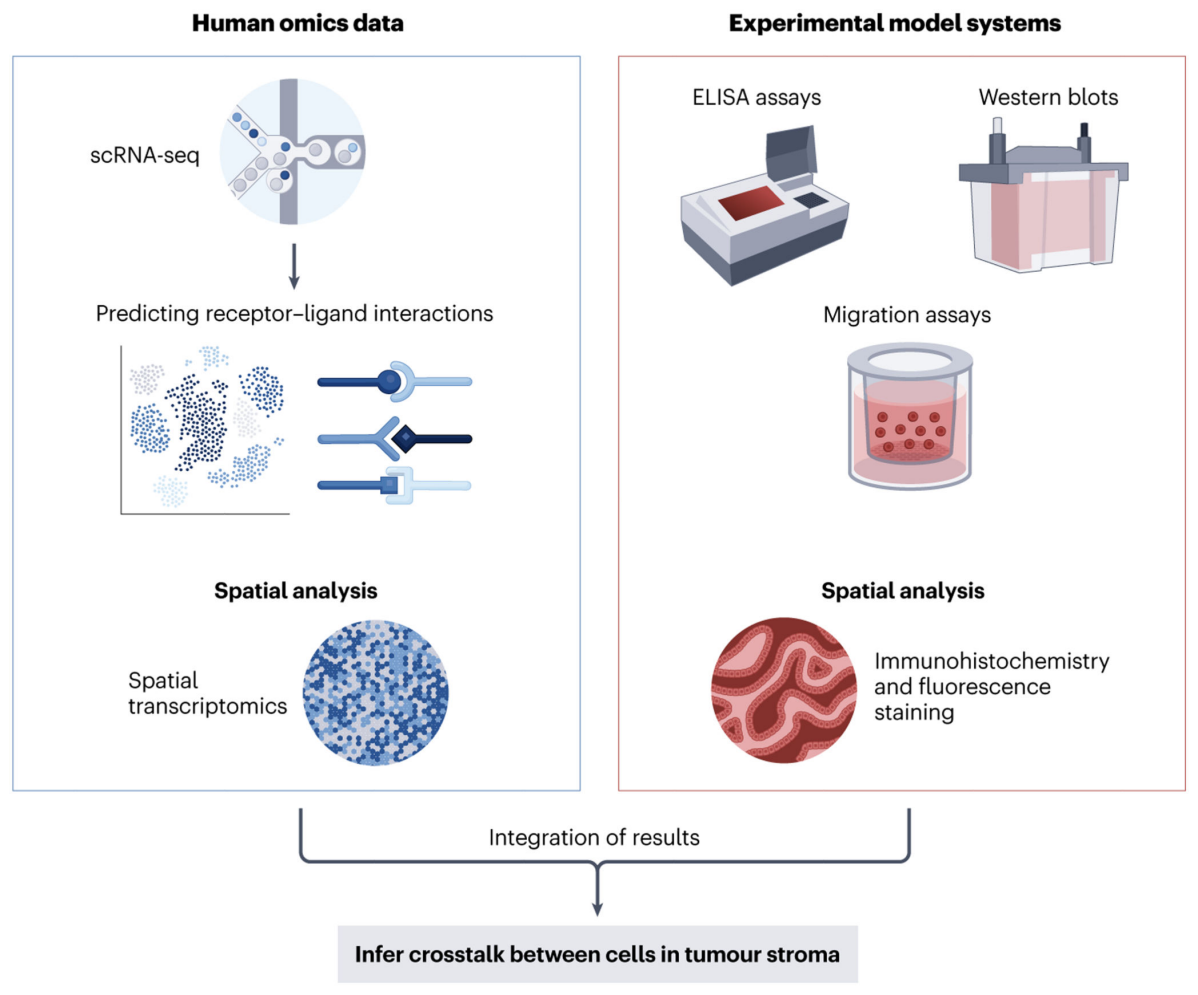
Inferences of cell crosstalk in the tumour stroma from experimental models and human omics data. The interactions between cells in the tumour stroma, including macrophages, neutrophils and fibroblasts, can be inferred through the analysis of single-cell RNA sequencing (scRNA-seq) data, whereby receptor–ligand binding pairs can be predicted. From functional experiments, techniques such as migration assays are often used to quantify the invasion of cells upon exposure to secreted factors, as determined by ELISA assays and Western blots. Advances in both fields now allow for the spatial architecture of the tumour microenvironment to be studied using immunohistochemistry and fluoresence staining, as well as spatial transcriptomics.

Interactions between cells in the tumour stroma can also have a role in promoting metastasis (Fig. 3). *In vitro* studies show that CAFs can promote the differentiation of monocytes into polarised macrophages with a pro-tumorigenic M2-like phenotype through the secretion of cytokines such as IL-6, IL-8 and IL-10^129,130^, many of which are also EMT-promoting factors. TAMs can also secrete IL-6, forming a positive-feedback loop that promotes further EMT and consequently increases metastasis efficacy^129,131^. Developments in omics technologies can now elucidate the crosstalk between cells in the stroma at a more granular level, building single-cell atlases^35^, investigating the interactions between, for example, specific subtypes of CAFs and TAMs, and highlighting important signalling axes and molecules involved^132,133^. This can be achieved through computational-based predictions of cell–cell communication from single-cell transcriptomic data by deriving receptor–ligand interactions^132,134^. Moreover, spatial omics technologies can add another layer of information through visualising cell positions and hence inferring likely interactions between cells^35,94,135^.

### Tumour cell dissemination

Upon intravasation, CTCs are key to the systemic dissemination of metastatic cancer through the blood^136^. CTCs can also occur in lymphatics, although less is known about lymph CTCs^137^. CTCs are observed in both patient blood samples and *in vivo* models — including mice with patient-derived xenografts — circulating as single cells or as clusters of up to 50 cells^138,139^. The latter, despite occurring at lower frequencies, are more favourable for dissemination, as they are more likely to resist apoptosis upon fluid shear stress and successfully seed a metastasis than are single cells^139,140^. CTC clusters are typically heterogeneous^138^; thus, the effects of intra-tumour heterogeneity and the presence of non-cancerous cells, such as TAMs, must be considered before the metastasis has even formed.

#### Heterogeneity of CTCs

Heterogeneity within CTCs can now be analysed using single-cell sequencing^141,142^. For example, in CTCs from a cohort of patients with breast cancer^143^, cells of both epithelial and mesenchymal phenotypes were found within individual clusters. Moreover, CTCs with a stem cell-like phenotype — simultaneously expressing both epithelial and mesenchymal markers — were detected. This is concordant with previous studies in mouse models and using patient data that identified CTCs co-expressing stem cell-like and epithelial and/or mesenchymal characteristics^141,144–146^. Transcriptomic studies have shown that CTCs and cells from the primary tumour have distinct transcriptional profiles^141^. In particular, CTCs have markedly decreased expression of genes involved in cell cycle progression and proliferation^147,148^. These genes, together with other oncogenes, were also subject to copy number alterations in CTCs; for example, *MYCN* was shown to undergo copy number amplifications in CTCs from breast cancer and lung cancer^149–151^. CTCs seem to have mutations and copy number profiles that are concordant with their subsequent metastases^151^, which suggests that CTC-inferred markers could be used as a non-invasive approach to predict the responses of patients with metastatic disease to different types of conventional treatment^145,148,150^.

#### Resistance of CTCs to shear stress

Despite their phenotypic adaptability, CTCs can be destroyed in the blood before reaching a metastatic site. One of the ways this might occur is through haemodynamic shear stress, from both the vessel walls and the circulating fluid itself, which induces anoikis **[G]** of CTCs^152,153^. CTCs can interact with nearby platelets, which can function as a physical ‘shield’ against both shear stress and the immune cells in circulation^154^. Bacteria can also act to dampen shear stress on CTCs. Using scRNA-seq, researchers have shown the enrichment of genes involved in responding to fluid shear stress in breast cancer organoids invaded by certain types of bacteria, including the commensal bacterium *Staphylococcus xylosus*^155^. Subsequent *in vivo* validation of these results also showed that bacterial invasion is associated with increased metastatic colonisation^155^. Nonetheless, within cell lines in an *in vitro* circulatory system, high levels of fluid shear stress were destructive towards cells of multiple cancer types, including breast, lung and ovarian cancers, whereas leukemic cells seemed to be more resistant to shear stress^153^. Interestingly, the surviving CTCs have more mesenchymal-like phenotypes downstream of JNK activation *in vitro*, which suggests that EMT is promoted during circulation^156^. Advances in sequencing techniques could provide further insights into the regulatory networks that modulate CTC morphology in the circulation before entering metastatic sites.

#### Mode of dissemination of tumour cells

Tumour cells can also disseminate without entering blood or lymph circulations^157^. For example, in a recent study of human lung adenocarcinoma that combined histopathological analysis with bulk sequencing, researchers found multiple metastatic lesions along the alveolar lining, indicating a lack of stromal invasion, which supports the hypothesis that tumours can spread through the airspaces^158^. Interestingly, the primary tumours that seeded metastases through the alveolar airspaces all had evidence of late dissemination, which was associated with disease recurrence and poor prognosis^158^. With the increasing amounts of omics data that are becoming available from large patient cohorts, it is likely that distinct and unique markers of different dissemination modes might be identified. In turn, this would aid the development of specific experimental models to, for example, better understand potential differences in the outcome of metastatic spread via different modes of dissemination.

#### Dormancy and reawakening of tumour cells

**Figure 4:**
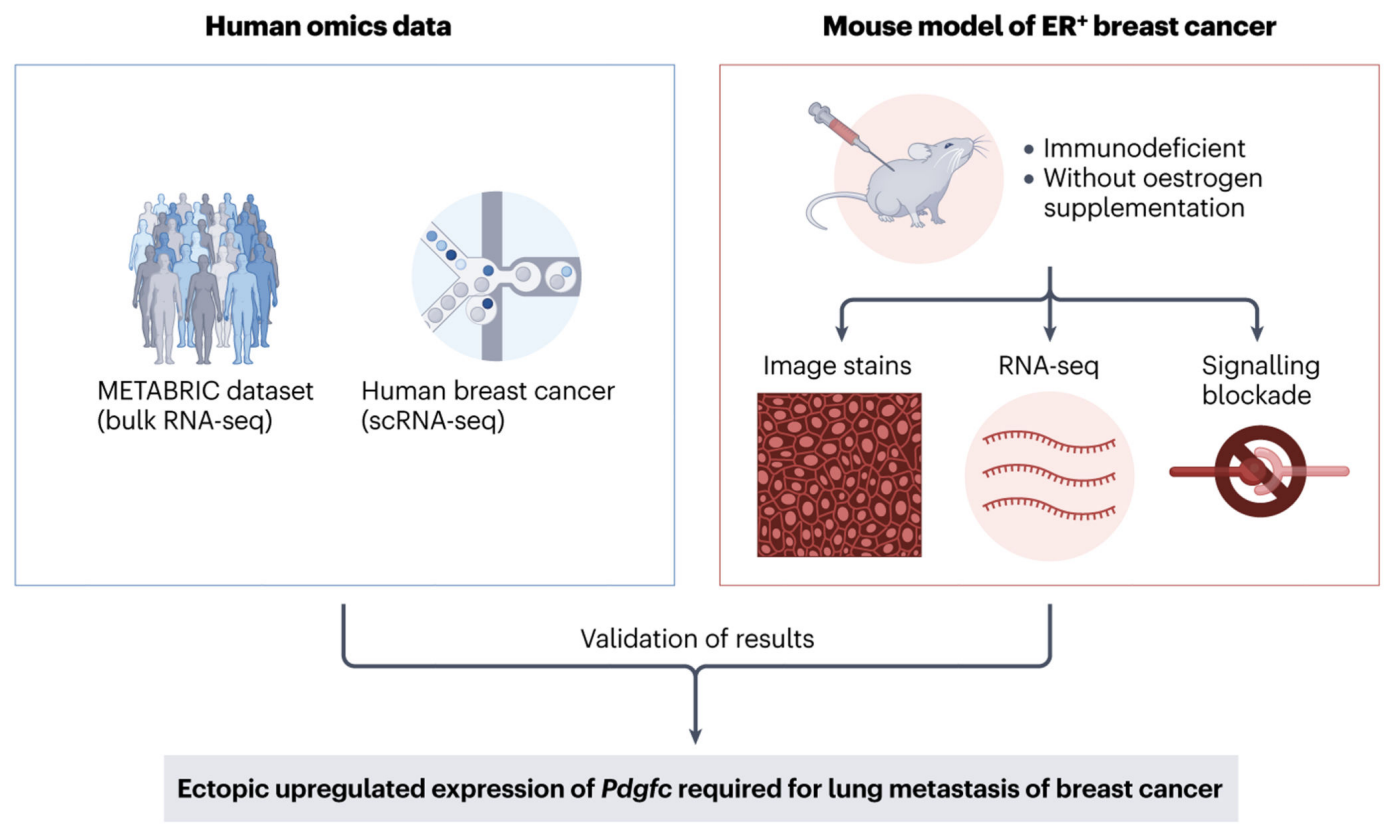
A case study for the integration of results from experimental models and human omics data. A recent study by Turrell et al^163^ used a mouse model of breast cancer to investigate the delayed onset of metastatic disease that is often observed in individuals with oestrogen receptor (ER)^+^ breast cancer. The model attempts to recapitulate the low oestrogen levels observed in older women but lacks a functional immune system. The authors used bulk RNA-sequencing (RNA-seq) data from the Molecular Taxonomy of Breast Cancer International Consortium (METABRIC) dataset, as well as single-cell RNA-seq (scRNA-seq) data from 26 human breast cancer samples to validate their finding in the mouse model that ectopic upregulation of *Pdgfc* in tumour cells is required for breast-to-lung metastasis.

Tumour cells that survive dissemination in the circulation can remain dormant in peripheral tissues and the bloodstream, leading to metastatic relapse months or years after initial surgery^159^. For example, breast cancer dormancy can last up to 20 years^160^. This poses many challenges for the study of metastasis in experimental models, both *in vitro*^161^ and *in vivo*^162^. However, progress has been made in deciphering how tumour cells reawaken after a period of dormancy. A recent study in oestrogen receptor (ER)^+^ breast cancer integrating results from mouse models and human omics data revealed increased ectopic expression levels of the growth factor PDGF-C in the TME of lung metastases in aged mice, which have accelerated metastatic outgrowth, compared with young mice, in which disseminated tumour cells have a dormant phenotype, or *in vitro* cell cultures^163^ (Fig. 4). Another *in vitro* model showed that neutrophil-mediated systemic inflammation expands a proliferative subpopulation of dormant mouse breast cancer cells in bone^164^. Although these results support the observation of late recurrence of disease occurring in individuals with previous breast cancer, improvements to such models should consider recapitulating biological and extrinsic factors that might affect the reawakening of dormant tumour cells, such as human hormone cycles and the increased use of hormonal therapies. The use of spatial omics technologies could also provide new insights in this area by revealing how localised interactions with neighbouring stromal and immune cells may contribute to the reawakening of dormant tumour cells. Meanwhile, longitudinal monitoring of patient populations with and without relapse may also enable further insights to these latter stages of the disease course.

## Conclusions and perspectives

From experimental model systems to the dawn of the multi-omics age, methods to study metastasis are developing rapidly. Here, we have explored how both fields have helped to elucidate key steps of the metastatic cascade and have highlighted how recent advances in the application of omics technologies to human tissue samples have supported and promoted long-standing conclusions from experimental systems. Reciprocally, biological models are being used to provide molecular insights to observations derived from large-scale sequencing studies of human tissue.

However, we must not overlook the limitations of both approaches. On the one hand, experimental systems of metastasis must faithfully recapitulate the human condition, or erroneous conclusions can be drawn. For example, although patient-derived xenograft models generally maintain the genetic and transcriptional diversity of the primary tumour and have been extensively used to predict drug responses with much success^165–167^, genomic variability has been observed between patient-derived xenograft models and the tumours from which they were derived^168^. This is likely due to genetic bottlenecking upon engraftment, which can result in discrepancies such as monoclonal models being generated from heterogeneous tumours^168^. On the other hand, a key limitation of the sequencing analysis of human metastatic samples has been a lack of longitudinal sampling. In this regard, autopsy studies could better represent the actual outcomes of metastatic dissemination in patients, although such data should be interpreted carefully in conjunction with each patient’s history of therapy, which can markedly alter the dynamics of dissemination and clonal evolution of metastases^33^. As recently reviewed^169^, such autopsy studies could also help to reduce the undeniably enormous logistical and financial efforts required for sample acquisition for sequencing studies. The co-recruitment of patients from large cohort studies such as TRACERx into autopsy studies such as PEACE (Posthumous Evaluation of Advanced Cancer Environment; a UK autopsy programme for patients with metastatic disease) could also allow for improved understanding of metastatic cancer progression and relapse from primary tumour development to death. Furthermore, the addition of other analysis methods, such as clinical imaging, will allow for a more comprehensive view of the interplay between metastases and other parts of the body; while genomic epidemiology could complement such work in metastasis research by revealing genetic risk factors of metastatic cancer in different populations

These two main approaches to metastasis research — experimental models and multi-omics analysis — must be used together to decipher the many remaining questions in the field. One of these is the effects of external, environmental stimuli — such as pollution, microplastics, diet, the microbiome and chronic stress — on the initiation, progression and reawakening of metastasis. For example, the onset of mental health disorders upon (metastatic) cancer diagnosis can negatively influence patient survival, through mechanisms including the induction of a more immunosuppressive microenvironment that consequently increases metastatic growth and progression^170,171^. Although exposure to these stimuli cannot be easily reduced and chronic stress caused by cancer cannot be simply prevented, it is important to acknowledge that these are disease-promoting factors that we, as a part of society, can conceivably control to some extent. Deepening our understanding of how such stimuli affect tumour growth and metastasis could help to control and reduce their impact. For example, would diet-led or antibiotic-induced changes in the gut microbiome alter metastatic progression? Could exposure to fine particulate matter during immunotherapy reduce treatment efficacy? How does physical exercise influence the metastatic transition? Answers to these questions can only be gained through the integration of all branches of metastasis research. For example, omics analyses of large datasets with comprehensive patient, geographic and socio-economic information could provide a holistic prediction of metastatic onset; subsequently, model systems could be used to elucidate markers and/or pathways underpinning the observations from omics studies in a repeatable and reproducible manner. In this way, we hope that results from future studies will not only lead to new ways in which to detect, prevent and treat cancer and metastases at the individual level, but also help to spearhead the implementation of policies and legislation for disease prevention at the population level.

## Glossary

### Abbreviations

Epithelial–mesenchymal transition: (EMT). The process by which an epithelial cell acquires mesenchymal characteristics, thereby increasing its invasiveness and migratory propensity towards metastasis.
Mesenchymal–epithelial transition: (MET). The reverse process of epithelial–mesenchymal transition, typically occurring in the later stages of metastasis (for example, during colonisation at a site distant from the primary tumour), whereby a migrating tumour cell with mesenchymal properties re-acquires epithelial characteristics.
Pre-metastatic niche: An environment distant from the primary tumour site that is primed for the colonisation and growth of a tumour metastasis.
Invadopodia: Finger-like protrusions of the plasma membrane of a cell that facilitate cell migration.
Circulating tumour DNA: (ctDNA). Fragments of DNA from tumour cells that can be detected in the bloodstream.
Matrix metalloproteinases: (MMPs). A family of proteolytic enzymes found in the extracellular matrix.
Anoikis: A form of apoptosis that occurs when cells detach from the extracellular matrix and/or neighbouring cells.
Basement membrane: A thin and pliable layer of extracellular matrix that lines tissue, providing structural support and separating cell types. Also known as basal lamina.
Triple-negative breast cancer: A fast-growing and invasive type of breast cancer lacking expression of HER2 and oestrogen and progesterone receptors.
Neoadjuvant therapy: Treatment provided prior to a main treatment. For cancer, examples include chemotherapy and hormone therapy; often used to shrink a tumour before surgery.
Confetti mouse: A genetically engineered mouse model which has a four-colour cassette that causes cells to be labelled randomly, allowing multicolour visualisation.
Contingent evolution: Evolution shaped by chance-based events, such as mutations.
Convergent evolution: Evolution of separate lineages in response to similar environmental pressures resulting in analogous phenotypes.
Table of Contents: This Review explores how experimental models of metastasis, such as mouse models and cell cultures, can complement the (multi-)omics analysis of human metastasis samples, thereby filling knowledge gaps left by model studies and validating the findings from human sequencing data.
